# Preparation of Foamed Ceramic from Cr Slag and MSWI Fly Ash and Its Cr Leaching Inhibition

**DOI:** 10.3390/ma18143372

**Published:** 2025-07-18

**Authors:** Hesong Li, Cheng Liu, Yikun Tang, Shilin Zhao

**Affiliations:** Hunan Engineering Research Center of Clean and Low-Carbon Energy Technology, School of Energy Science and Engineering, Central South University, Changsha 410083, China; lihesong@csu.edu.cn (H.L.); 223912077@csu.edu.cn (C.L.); 243912086@csu.edu.cn (Y.T.)

**Keywords:** Cr slag, MSWI-FA, foamed ceramic, Inhibitors, Cr leaching

## Abstract

The sustainable utilization of solid waste is crucial for environmental protection. This work investigates the fabrication of foamed ceramics from Cr slag and municipal solid waste incineration (MSWI) fly ash, focusing on the effects of three inhibitors—NH_2_SO_3_H, ZnO·TiO_2_, and (NH_4_)_2_HPO_4_—on material properties and Cr leaching behavior. Experimental analysis, chemical thermodynamic calculations, and material characterization were all employed. Results show that the prepared foamed ceramics meet the JG/T 511-2017 standard for building materials, exhibiting excellent physical properties but significant Cr leaching. Among the inhibitors, (NH_4_)_2_HPO_4_ with a molar ratio of n(P)/n(Cr) = 1 shows the best performance, achieving a bulk density of 205 kg/m^3^, compressive strength of 0.850 MPa, Cr leaching concentration of 188 μg/L, and a 70.0% of Cr leaching inhibition rate. The improvement is attributed to the AlPO_4_ formation that enhancing the strength, and Ca_2_P_2_O_7_ that stabilizing Cr during sintering. This work provides a feasible method for the safe resource utilization of Cr-containing waste.

## 1. Introduction

### 1.1. Research Background

Cr slag and municipal solid waste incineration fly ash (MSWI-FA) are two major types of hazardous solid waste produced in large quantities in China. As the world’s largest producer and consumer of sodium dichromate [1], China generates approximately 2.50 × 10^5^ tons of Cr slag annually, with a historical accumulation of nearly six million tons [2,3,4]. Cr slag contains Cr(VI), a highly mobile, toxic, and carcinogenic substance that poses significant threats to human health and the environment [5,6]. Meanwhile, MSWI-FA, a byproduct of the widely adopted waste-to-energy technology with over 10 million tons of that generated annually, presents similar environmental concerns [2,3,4]. Therefore, developing effective treatment and utilization methods for these wastes has become an urgent and enormous challenge.

### 1.2. Current Research Status and Limitations

In recent years, studies on the potential of utilizing Cr slag and MSWI-FA as raw materials for resource recovery have been explored. Liu et al. [7] prepared ceramic with a water absorption of 0.08% and a flexural strength of 124.78 MPa based on the SiO_2_-Al_2_O_3_-CaO-MgO (8 wt.%) phase diagram using different types of MSWI-FA and other solid wastes. Hou et al. [8] produced tiles using Cr slag and copper smelting waste slag, achieving a compressive strength of 127.2 MPa and a Cr (VI) leaching concentration of 3.31 mg/L, with a Fe/Cr ratio of 1.5, a sintering temperature of 1200 °C, and a holding time of 30 min. Hou et al. [9] utilized Cr slag as the main raw material to fabricate black ceramic tiles, achieving a compressive strength of 162.61 MPa and a Cr(VI) leaching concentration of 0.97 mg/L under optimal conditions (Fe/Cr/Mn molar ratio of 3:2:1, sintering at 1150 °C for 45 min). These studies achieve the resource conversion and utilization of waste Cr slag.

Among various solid waste-derived products, foamed ceramics have attracted increasing attention due to their excellent properties such as low density, thermal insulation, corrosion and oxidation resistance. It is often referred to as “solid waste gold”, which has emerged as a major research hotspot in the field of solid waste utilization, Ge at al. [10] developed closed-cell foam ceramic by using Cr slag and coal bottom ash as raw materials. With 30 wt.% Cr slag, the ceramic achieved a bulk density of 0.191 g/cm^3^, a compressive strength of 1.3 MPa, a pore diameter of 0.73 mm, and a Cr leaching concentration of 0.004 mg/L. Xu et al. [11] adopted Cr slag as the primary raw material for synthesizing microcellular foamed ceramics, with copper oxide and manganese oxide incorporated as additives. It facilitated the reuse of Cr slag, and made the foamed ceramics with enhanced thermal insulation properties. Liu et al. [12] utilized Fe-Cr slag, industrial alumina, and silica powder as raw materials to produce porous cordierite ceramic with a flexural strength of 47.26 ± 1.01 MPa, a coefficient of thermal expansion of 3.5 × 10^−6^/°C, and a Cr leaching rate of 35.0 mg/kg.

However, high-temperature treatment often induces oxidation of Cr(III) to Cr(VI), significantly increasing Cr leaching risk. Additionally, alkali metals and alkaline earth metals, such as Ca, Na, and K, are the key factors leading to Cr leaching during Cr slag incineration or other thermal treatments [13,14]. Gao et al. [15] used sulfates (NH_2_SO_3_H, NH_4_HSO_4_, NaHSO_4_) to mitigate Cr leaching during tannery sludge incineration, which found the reaction between sulfate and CaO effectively inhibited Cr leaching, with NH_2_SO_3_H showing the most significant effect. Nearly all Ca was bound to the CaSO_4_ phase, which retained the ability to oxidize Cr_2_O_3_ to CaCrO_4_. Zhao et al. [16] found adding ZnO or TiO_2_ reduced Cr leaching during the heat treatment of Cr-containing tannery sludge, where addition of ZnO·TiO_2_ produced a notably inhibitory effect. Mao et al. [17] demonstrated (NH_4_)_2_HPO_4_ could reduce Cr through chemical thermodynamic calculation, which achieved a detoxification rate of over 97% after heat treatment. It can be found that NH_2_SO_3_H, ZnO·TiO_2_, and (NH_4_)_2_HPO_4_ are effective in inhibiting Cr leaching for Cr-containing solid wastes during thermal treatment. However, there are no reports on the effects of these inhibitors on the physical and Cr leaching properties of foamed ceramic prepared from Cr slag and MSWI-FA (CS-MSWI-FA-FC). The optimal inhibitor and its ratio, and related inhibition mechanism need to be explored.

### 1.3. Main Content and Objectives

In this study, the influence of inhibitor types (NH_2_SO_3_H, ZnO·TiO_2_, and (NH_4_)_2_HPO_4_) and additional amount on the physical properties and Cr leaching behavior of CS-MSWI-FA-FC was systematically investigated. Combining the thermal treatment experiments, acetic acid buffer leaching tests (HJ/T 300-2007 [18]), characterization techniques (scanning electron microscopy (SEM), X-ray fluorescence spectroscopy (XRF), X-ray diffraction (XRD)), and chemical thermodynamic simulation (HSC Chemistry software, https://www.hsc-chemistry.com/), the influencing mechanism on the physical and Cr leaching of the CS-MSWI-FA-FC were systematically explored. It aims to obtain the optimal type and additional amount of inhibitors, and to reveal the inhibition mechanism, which hopes to provide technical support and theoretical guidance for the synergistic resource utilization of Cr slag and MSWI fly ash in the preparation of foamed ceramics.

## 2. Materials and Methods

### 2.1. Preparation Process of CS-MSWI-FA-FC

**Figure 1:**
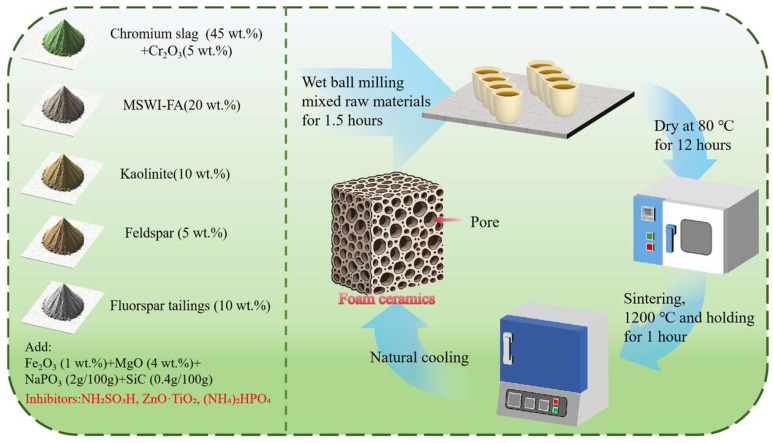
Preparation process of CS-MSWI-FA-FC.

The preparing process of CS-MSWI-FA-FC is illustrated in Figure 1. The procedure involved weighing specified proportions of solid wastes, including Cr slag and MSWI-FA, etc., adding MgO, Fe_2_O_3_, and other materials to achieve the desired compositional balance for the foamed ceramic system. To study Cr leaching, three inhibitors including NH_2_SO_3_H, ZnO·TiO_2_, and (NH_4_)_2_HPO_4_ were introduced at comparable molar ratios. A small amount of SiC was added as a foaming agent. The solid–liquid volume ratio was set to 1:2, and the mixture was ball-milled for 1.50 h to ensure thorough homogenization. After drying, the material was sieved through a 60.0-mesh screen, poured into a high-temperature-resistant crucible, and placed in a muffle furnace. The temperature setting program was 25.0–600 °C for 1.00 h; 600–950 °C for 80 min; 950–1.20 × 10^3^ °C for 90.0 min; and then maintained at 1.20 × 10^3^ °C for 1.00 h and cooled naturally to room temperature. The final product was the CS-MSWI-FA-FC.

### 2.2. Toxicity Leaching Experiment

The leaching characteristics of Cr from the foamed ceramic were assessed using the acetic acid buffer solution method specified in *HJ/T 300-2007* [18]. An acetic acid solution with a pH of 2.64 ± 0.05 was employed as the leaching solution, with a solid–liquid ratio of 1:20 (g/mL). The mixture was then oscillated on a flip-type oscillating device at 30 ± 2 r/min for 18.0 h. The Cr concentration in the leachate was determined using inductively coupled plasma optical emission spectrometer (ICP-OES, SPECTRO BLUE SOP, Kleve, Germany).

To further evaluate the inhibition effect of Cr leaching, the inhibition rate of Cr, denoted as P (%), was introduced, in addition to the Cr leaching concentration, as shown in Equation (1).

(1) $$P=\frac{C_{x}-C_{0}}{C_{0}}\times 100\%$$

where C_0_ denotes the leaching concentration of Cr in the raw sample (μg/L), and C_x_ (where x represented NH_2_SO_3_H, ZnO·TiO_2_, or (NH_4_)_2_HPO_4_) denotes the leaching concentration of Cr in the sample after adding the corresponding inhibitor (μg/L).

### 2.3. Chemical Thermodynamic Calculation

The chemical thermodynamics software named HSC Chemistry was used to calculate the reactions within the system. Based on the XRF results of Cr slag, MSWI-FA, and other solid wastes, the main components of the reaction inputs for the thermodynamic calculations were Na_2_O, MgO, Al_2_O_3_, SiO_2_, CaO, K_2_O, Fe_2_O_3_, Cr_2_O_3_, as well as ZnO and TiO_2_. Since (NH_4_)_2_HPO_4_ and NH_2_SO_3_H decomposed easily at high temperature into equivalent amount of H_3_PO_4_ and H_2_SO_4_, they were used as replacement inputs in the calculation. Thermodynamic data for some reaction products were supplemented from the Refs. [19,20,21]. The possible Cr forms in the reaction products are shown in Table 1. Considering the reactants and reaction conditions, the possible products of elemental P in the (NH_4_)_2_HPO_4_ inhibitor included Ca(PO_3_)_2_, Ca_2_P_2_O_7_ [22], Na_3_PO_4_, and K_3_PO_4_ [23], etc. The possible products of elemental S in the NH_2_SO_3_H inhibitor were Cr2(SO_4_)_3_, CaSO_4_, SO_2_(g) [15], etc.

### 2.4. Testing and Characterization of Foamed Ceramic Properties

The prepared ceramic samples were analyzed by XRD (Rigaku SmartLab SE, Tokyo, Japan) to determine their main mineral phases under Cu Kα radiation, with a 2θ range of 5–90° and a step size of 0.02°. The bulk density of the product was measured using the Archimedes drainage method, with water as the buoyant medium. The sample mass was determined using an analytical balance with a sensitivity of 10^−4^ g [27]. The SEM (TESCAN MIRA LMS, Shanghai, China) was used to observe the surface morphology of the foamed ceramic at various scales. The compressive strength and compressive modulus of the foamed ceramic were measured by using an electronic universal testing machine (MTS System CMT6103, Shanghai, China) with an entrance force of 1.00 N and a tensile rate of 10.0 mm/min. According to the performance requirements for foamed ceramic insulation board II_w_ without an axial surface in *JG/T 511-2017 Foamed Ceramic Thermal Insulation Board in Building* [28], the bulk density should be between 180 and 230 kg/m^3^, and the compressive strength should be ≥0.500 MPa.

## 3. Results and Discussion

### 3.1. General Information of Cr Slag, MSWI-FA, and Other Solid Wastes

The XRF results of Cr slag, MSWI-FA, and other solid wastes are presented in Table 2. Cr slag primarily consists of SiO_2_, Al_2_O_3_, and Fe_2_O_3_, with Cr_2_O_3_ as the main Cr form. To enhance the representativeness of the experimental results, pure Cr_2_O_3_ is added to the Cr slag, increasing its total content to 10.0 wt.% [29]. The MSWI-FA, dried after washing, mainly contains CaO, SiO_2_, and small amounts of MgO and Fe_2_O_3_. Kaolin, feldspar, and fluorspar tailings are predominantly composed of SiO_2_. In addition, kaolin and feldspar contain high contents of Na_2_O and K_2_O, which play a fluxing role in the preparation of foamed ceramic.

### 3.2. Physical and Cr Leaching Properties of CS-MSWI-FA-FC Without Inhibitors

**Figure 2:**
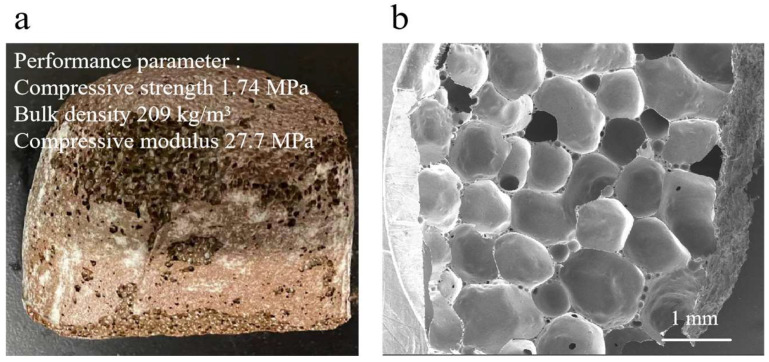
CS-MSWI-FA-FC (without inhibitors): (**a**) physical properties; (**b**) SEM images.

The CS-MSWI-FA-FC (without inhibitors) and their corresponding SEM images are shown in Figure 2. It shows the CS-MSWI-FA-FC exhibit a well-developed pore structure without inhibitor. The compressive strength, bulk density, and compressive modulus are 1.74 MPa, 209 kg/m^3^, and 27.7 MPa, respectively, which meet the performance requirements of *JG/T 511-2017 building material standard* [28]. This indicates their potential to be used in the production of foamed ceramic thermal insulation boards for buildings.

The microstructure of the prepared foamed ceramic is shown in Figure 2b, which reveals the pore distribution and size are uniform, with a pore diameter of approximately 1 mm. This uniformity ensures both the mechanical strength and thermal insulation performance. SiC powder is commonly used as a blowing agent in the foamed ceramic producing. At temperatures above 900 °C, SiC typically undergoes an oxidation reaction, as shown in Equation (2). The toxic leaching of Cr from the prepared foamed ceramic is 628 μg/L, which complies with the *GB 16889-2008 Standard for Pollution Control on the Landfill Site of Municipal Solid Waste* [30]. However, there is still potential for further reduction in its leaching.

(2) $$\mathrm{SiC}+O_{2}\text{→}\mathrm{Si}O_{2}+CO_{2}\text{↑}+\mathrm{CO}\text{↑}$$

### 3.3. Effects of Inhibitor Types on the Physical and Cr Leaching Properties

**Figure 3:**
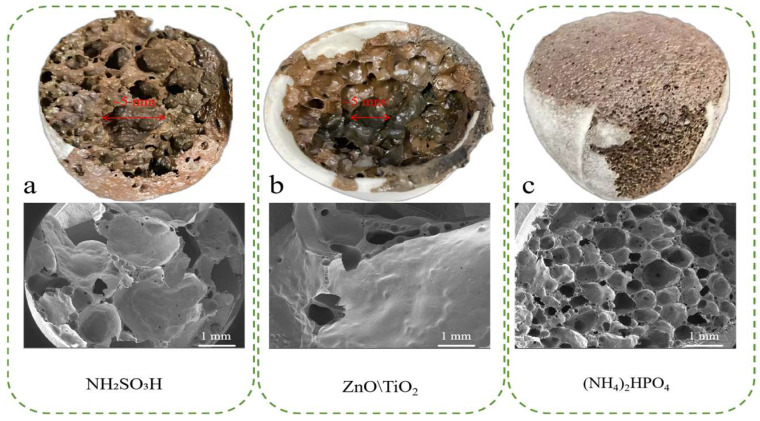
CS-MSWI-FA-FC after adding inhibitors and their corresponding SEM images. (**a**) NH_2_SO_3_H; (**b**) ZnO·TiO_2_; (**c**) (NH_4_)_2_HPO_4_.

Three inhibitors, namely, NH_2_SO_3_H, ZnO·TiO_2_, and (NH_4_)_2_HPO_4_, are selected based on previous studies. The ratios for the inhibitors are as follows: NH_2_SO_3_H: n(S)/n(Cr) = 2 [15]; ZnO·TiO_2_: n(Zn)/n(Cr) = 1, n(Ti)/n(Ca) = 0.8 [31]; and (NH_4_)_2_HPO_4_: n(P)/n(Cr) = 2 [17]. The prepared samples after adding inhibitors and their corresponding SEM images are shown in Figure 3. It shows the pore distribution in the foamed ceramic prepared with NH_2_SO_3_H and ZnO·TiO_2_ is not uniform, with some pores exceeding 5 mm. While other smaller pores, approximately 1 mm, are also present. This may be attributed to the significant difference between foaming temperature and formation temperature of the molten phase with the addition of these inhibitors. As a result, the molten phase in the sample is insufficient to encapsulate the gas generated by the blowing agent, leading to the small bubbles rising and eventually merging into larger bubbles [32]. In contrast, the overall pore distribution in the samples prepared with (NH_4_)_2_HPO_4_ is uniform, with small pores ranging from 0.2 to 1 mm.

**Figure 4:**
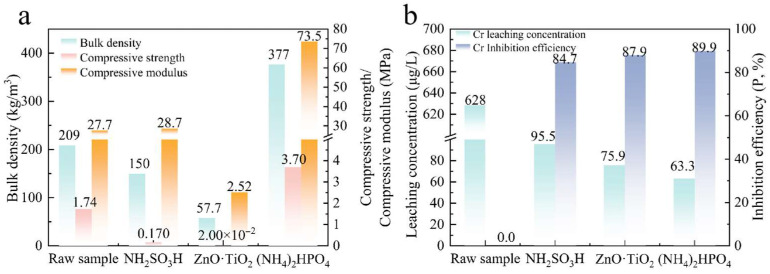
Physical properties and Cr leaching characteristics of CS-MSWI-FA-FC prepared with different inhibitors: (**a**) physical properties; (**b**) Cr leaching properties.

The physical properties of the CS-MSWI-FA-FC prepared with different inhibitors are shown in Figure 4a. The structural properties of the ceramic material deteriorate after adding NH_2_SO_3_H, resulting in a bulk density of 150 kg/m^3^ and a compressive strength of only 0.170 MPa. This may be attributed to two factors: (1) the pores within the ceramic structure become uneven, with an increase in pore diameter and a reduction in pore wall thickness; and (2) the sulfate formed during the process decomposes at high temperature, producing SO_2_, which increases internal gas pressure and promotes bubble coalescence [33]. The compression modulus remains relatively unchanged at 28.7 MPa, indicating that the addition of NH_2_SO_3_H has minimal effect on the material’s plasticity. After the addition of ZnO·TiO_2_, the bulk density, compressive strength, and compressive modulus decrease to 57.7 kg/m^3^, 2.00 × 10^−2^ MPa, and 2.52 MPa, respectively, resulting in the inability to form a ceramic material with sufficient structural strength. This may be because the addition of ZnO·TiO_2_ changes the main components of the system, resulting in large differences in foaming temperature and formation temperature of the molten phase, and the bubbles become larger [10]. After adding (NH_4_)_2_HPO_4_, the volume density is 377 kg/m^3^, the pores are smaller, and the volume density is nearly doubled compared to the raw sample. The compressive strength and compression modulus are significantly increased to 3.70 MPa and 73.5 MPa, respectively. This shows the structural strength of the samples is stronger and the deformation degree is smaller when squeezed after adding (NH_4_)_2_HPO_4_. This may be because the generated phosphate has a higher density and also has a higher compressive strength at room temperature [34,35]. In summary, the addition of NH_2_SO_3_H and ZnO·TiO_2_ results in incomplete sample forming, uneven bubbles and poor material properties. However, the sample obtained after adding (NH_4_)_2_HPO_4_ is more in line with the standard of foamed ceramic insulation board for construction in terms of material performance indicators, the pores are more uniform and the compressive strength is more advantageous.

The Cr leaching characteristics of the CS-MSWI-FA-FC prepared with different inhibitors are shown in Figure 4b. Compared to the foamed ceramic without adding inhibitors (raw sample), all three inhibitors exhibit better inhibition on Cr leaching, with the Cr leaching concentration below 100 μg/L and inhibition rate exceeding 80.0%. Among the inhibitors, NH_2_SO_3_H demonstrates the least inhibition effects, with a Cr leaching concentration of 95.5 μg/L and an inhibition rate of 84.7%. (NH_4_)_2_HPO_4_ has the best inhibitory effect on the CS-MSWI-FA-FC, with a Cr leaching concentration of 63.3 μg/L and an inhibition rate of nearly 89.9%. Considering the physical properties of foamed ceramic and the inhibitory effect on Cr leaching, (NH_4_)_2_HPO_4_ is the best inhibitor.

### 3.4. Effect of (NH_4_)_2_HPO_4_ Amount on the Physical and Cr Leaching Properties

**Figure 5:**
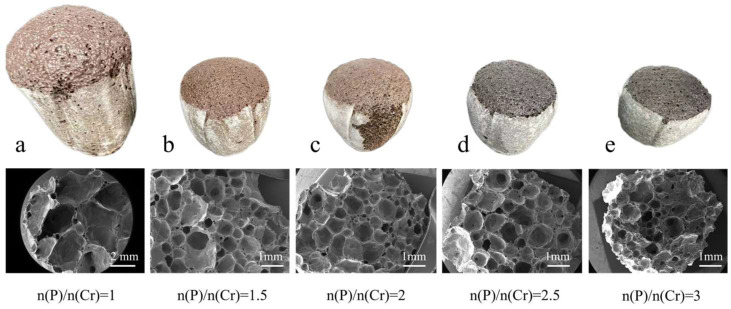
CS-MSWI-FA-FC prepared with different additional amounts of (NH_4_)_2_HPO_4_ and their corresponding SEM results. (**a**) n(P)/n(Cr) = 1; (**b**) n(P)/n(Cr) = 1.5; (**c**) n(P)/n(Cr) = 2; (**d**) n(P)/n(Cr) = 2.5; (**e**) n(P)/n(Cr) = 3.

The CS-MSWI-FA-FC prepared with different additional amounts of (NH_4_)_2_HPO_4_ and their corresponding SEM results are shown in Figure 5. With the increase in added (NH_4_)_2_HPO_4_ quantity, the volume of the samples gradually decreases. The color of the foamed ceramic gradually changes from brown to black (the white substance on the surface is the high-temperature resistant aluminum silicate fiber between the material and the crucible during sintering). The SEM results show that the pore size reaches 2 mm and the pore distribution is uniform when n(P)/n(Cr) is 1. When n(P)/n(Cr) ≥ 1.5, the pore size decreases are all less than 1 mm, and the small pores on the pore wall are larger. This may be due to the difference between the melting phase and foaming temperature, which causes the pores to not merge during the foaming process.

**Figure 6:**
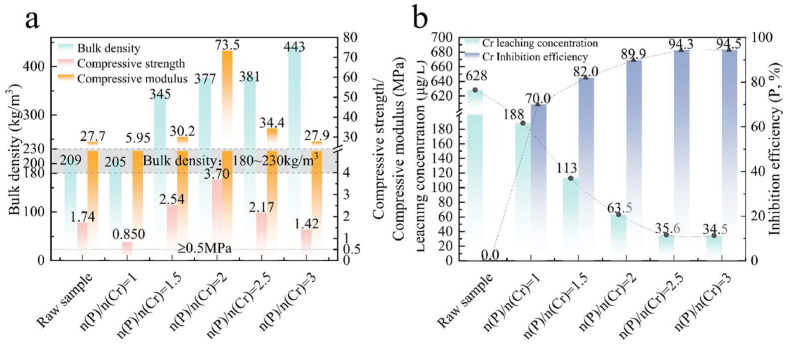
Physical properties and Cr leaching characteristics of CS-MSWI-FA-FC prepared with different additional amounts of (NH_4_)_2_HPO_4_: (**a**) Physical properties; (**b**) Cr leaching characteristics.

The physical properties of the samples with different additional amounts of (NH_4_)_2_HPO_4_ are shown in Figure 6a. Compared with the raw sample, the volume density gradually increases with the increase in quantity of additional (NH_4_)_2_HPO_4_, indicating the pores gradually decrease, which is consistent with Figure 5. Compared with the raw sample and n(P)/n(Cr) = 1, the compressive strength decreases slightly from 1.74 MPa with no addition to 0.850 MPa, and the compression modulus also decreases from 27.7 MPa to 5.95 MPa accordingly. The material properties at this condition still meet the *JG/T511-2017* standard [28]. When n(P)/n(Cr) further increases, the compressive strength and compression modulus firstly increase and then decrease, reaching a peak value of 3.70 MPa and 73.5 MPa, respectively, at n(P)/n(Cr) = 2. The addition of (NH_4_)_2_HPO_4_ may cause the oxygen bonds in the SiO_2_ structure to dissociate after sintering at 1.20 × 10^3^ °C, which in turn leads to the transformation of a small amount of quartz to cristobalite. It will lead to volume expansion, density reduction, and deterioration of mechanical properties of SiO_2_ [35]. However, the addition of (NH_4_)_2_HPO_4_ may lead to the formation of phosphates during the preparation, which is beneficial to improving its compressive strength and compression modulus. Therefore, the volcano shape changes in compressive strength and compression modulus with the increase in n(P)/n(Cr) is the game result between these two reasons.

Cr leaching characteristics of CS-MSWI-FA-FC prepared with different additional amounts of (NH_4_)_2_HPO_4_ is shown in Figure 6b. As the additional amount of (NH_4_)_2_HPO_4_ increases, the Cr leaching concentration gradually decreases and the inhibition rate gradually increases. When n(P)/n(Cr) = 1, the Cr leaching concentration and inhibition rate is 188 μg/L, and 70.0%, respectively, while they are 35.6 μg/L, and 94.3% at n(P)/n(Cr) = 2.5. As the amount of (NH_4_)_2_HPO_4_ increases further, the Cr leaching does not change significantly. The addition of (NH_4_)_2_HPO_4_ has a good inhibitory effect on Cr leaching. Considering the physical properties and the Cr leaching characteristics of the CS-MSWI-FA-FC, the optimal additional amount of (NH_4_)_2_HPO_4_ is n(P)/n(Cr) = 1.

### 3.5. Mechanism Analysis

**Figure 7:**
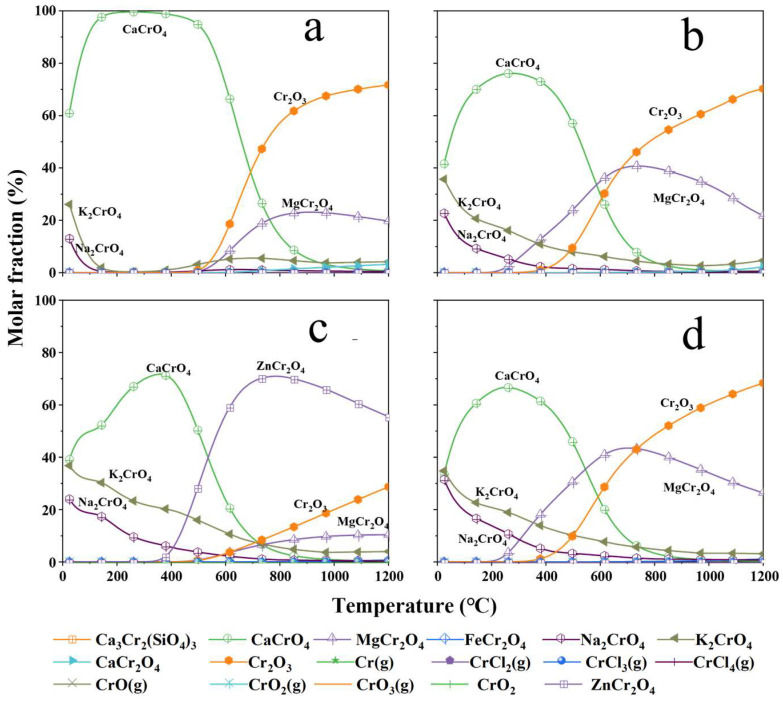
Chemical thermodynamic calculation results at various temperatures within the sintered system for the CS-MSWI-FA-FC and that with three inhibitors. (**a**) Raw sample; (**b**) NH_2_SO_3_H; (**c**) ZnO·TiO_2_; (**d**) (NH_4_)_2_HPO_4_.

The chemical thermodynamic calculation results at different temperatures in the sintering system of the raw sample and that with three inhibitors are shown in Figure 7. It shows that the Cr in the raw sample is easily oxidized to form Cr (VI) (CaCrO_4_, K_2_CrO_4_, Na_2_CrO_4_) at ≤1000 °C [17]. However, a small amount of Cr (III) (CaCr_2_O_4_) is generated under a high temperature, which is prone to Cr leaching [26]. In this work, the Cr forms are mainly Cr_2_O_3_ and MgCr_2_O_4_ when the temperature rises, which are not easy to leach with good Cr stability. For CaCrO_4_, the inhibition of Cr (VI) occurs at a lower temperature, while the Cr form does not change significantly at high temperature after adding NH_2_SO_3_H and (NH_4_)_2_HPO_4_. After adding ZnO·TiO_2_, the Cr morphology changes significantly at temperature above 400 °C, which generates a spinel phase (ZnCr_2_O_4_) with poor Cr leaching. It is consistent with the experimental results of Zhao et al. [16] and Yang et al. [31].

**Figure 8:**
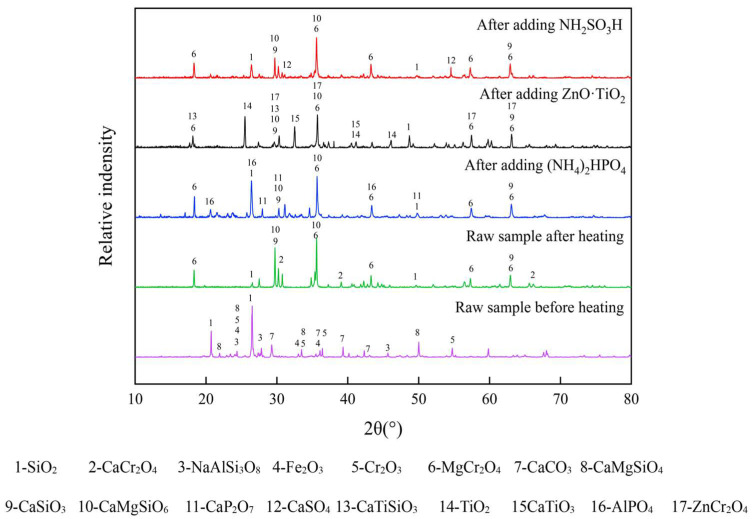
XRD results of the raw sample before and after sintering, and after sintering with the inhibitors.

The XRD results of the raw sample before and after sintering, and that after sintering with different inhibitors, are shown in Figure 8. The main components are SiO_2_, CaCr_2_O_4_, MgCr_2_O_4_, CaSiO_3_, CaMgSiO_6_, after sintering of the raw sample, where CaCr_2_O_4_ is the main Cr leaching substance. MgO can combine with Cr to form spinel phase MgCr_2_O_4_, which is consistent with the chemical thermodynamic calculation results. It has a certain Cr fixation effect [36]. According to the ionic theory of slag, the diffusion degree between the reactants is determined by the activity of ion transfer. The more alkaline substances there are, the more O^2−^ ions are ionized, and the greater the probability of Cr being oxidized. In this work, CaO and SiO_2_ are alkaline and acidic substances, respectively. Therefore, SiO_2_ mainly combines with CaO to form CaSiO_3_ to prevent it from participating in the oxidation reaction of Cr in the raw sample.

**Figure 9:**
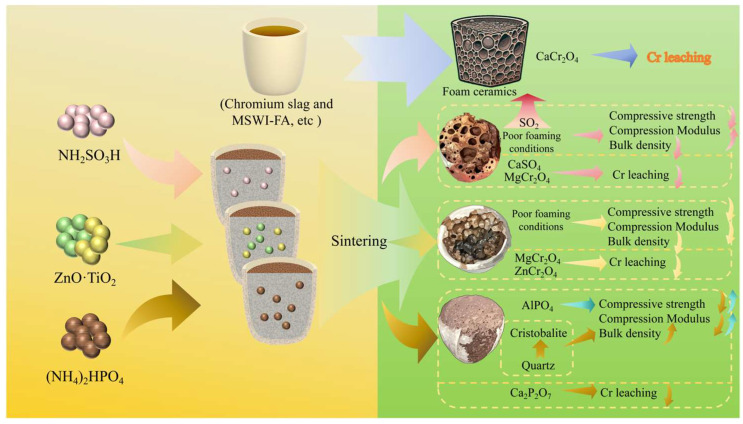
Influence mechanism of three inhibitors on the physical and Cr leaching properties of CS-MSWI-FA-FC.

In addition to the main substances of the raw sample after sintering, the respective addition of NH_2_SO_3_H, ZnO·TiO_2_, and (NH_4_)_2_HPO_4_ also increases some substances, such as CaSO_4_, ZnCr_2_O_4_, CaTiSiO_5_, CaTiO_3_, Ca_2_P_2_O_7_, and AlPO_4_. NH_2_SO_3_H and (NH_4_)_2_HPO_4_ can combine with alkaline oxides to generate CaSO_4_ and Ca_2_P_2_O_7_. The acidity of H_2_SO_4_ is stronger than that of H_3_PO_4_. NH_2_SO_3_H has a stronger ability to inhibit Cr leaching in theory. However, the experimental result shows (NH_4_)_2_HPO_4_ has a better inhibitory effect. It may be that CaSO_4_ decomposes at 1.20 × 10^3^ °C to produce SO_2_ and produces free Ca^2+^, which will further form CaCr_2_O_4_ that is easy to leach [17]. Meanwhile, the generated SO_2_ may be the main reason for the pore increased in the sample [33]. ZnO, as a divalent metal oxide, can combine with trivalent metal oxides such as Cr_2_O_3_ to form a spinel phase. TiO_2_ is an amphoteric metal oxide, so it can also combine with CaO to form CaTiO_3_, which thereby fixes Ca and inhibits Cr leaching. Judging from the inhibition effect of Cr leaching, (NH_4_)_2_HPO_4_ has stronger reducing properties compared to SiO_2_ in the raw sample [17]. Moreover, the liquid H_3_PO_4_ decomposed from (NH_4_)_2_HPO_4_ increases the contact area between CaCrO_4_ and phosphate and promotes Ca_2_P_2_O_7_ formation. Therefore, increasing the additional amount of (NH_4_)_2_HPO_4_ can significantly inhibit the Cr leaching [22]. From the physical properties of CS-MSWI-FA-FC, adding (NH_4_)_2_HPO_4_ can significantly affect the pore structure, where the generated phosphate AlPO_4_ will significantly improve the compressive strength that meets the building material standard. The influence mechanism of three inhibitors on the physical and Cr leaching properties of CS-MSWI-FA-FC is summarized in Figure 9.

## 4. Conclusions

The foamed ceramic prepared by Cr slag and MSWI-FA exhibit an (excellent) compressive strength, bulk density and compression modulus of 1.74 MPa, 209 kg/m^3^ and 27.7 MPa, respectively, meeting the *JG/T 511-2017 building materials standard*. However, the Cr leaching is relatively high at 628 μg/L, which is mainly caused form CaCr_2_O_4_. (NH_4_)_2_HPO_4_ has the best inhibitory effect on Cr leaching of the CS-MSWI-FA-FC among the three inhibitors. As the additional amount of (NH_4_)_2_HPO_4_ increases, the volume density gradually increases, the compressive strength and compression modulus have volcano-shaped changes, and the inhibition effect of Cr leaching gradually improves. Considering the physical requirements of foamed ceramic and Cr leaching, (NH_4_)_2_HPO_4_ and n(P)/n(Cr) = 1 are the best inhibitor type and additional amount. The difference between the temperatures of the molten phase and the foaming affects the foaming process and the final physical properties. (NH_4_)_2_HPO_4_ will promote the formation of phosphate AlPO_4_, etc., thereby improving the compressive strength. It also has a stronger reducibility and combines with Ca to form Ca_2_P_2_O_7_ during sintering to inhibit Cr leaching.

In future work, it is necessary to consider the long-term Cr leaching stability of foamed ceramics, and evaluate their environmental impact and economic feasibility from a life cycle perspective, so as to provide guidance for the industrial application of the resource utilization technology of foamed ceramics prepared with Cr slag and MSWI fly ash.

## Figures and Tables

**Table 1:** Possible Cr forms in the reaction products.

Phase	Components	Ref.
Gas phase	Cr, CrO, CrO_2_, CrO_3_, CrCl_4_, CrCl_3_, CrCl_2_	[24,25]
Solid phase	Ca_3_Cr_2_(SiO_4_)_3_, CaCrO_4_, CaCr_2_O_4_, Cr_2_O_3_, MgCr_2_O_4_, K_2_CrO_4_, Na_2_CrO_4_, CrO_2_, FeCr_2_O_4_, Cr_2_(SO_4_)_3_	[15,25,26]

**Table 2:** XRF results of Cr slag, MSWI-FA, and other solid wastes (wt.%).

	Na_2_O	MgO	Al_2_O_3_	SiO_2_	Cl	K_2_O	CaO	TiO_2_	Cr_2_O_3_	Fe_2_O_3_	Others
Cr slag	1.32	1.90	15.4	62.6	0.0500	2.60	0.670	0.560	0.0700	14.1	0.750
MSWI-FA	0.920	2.18	1.63	9.55	0.0700	0.560	72.8	0.310	0.290	1.39	10.4
Kaoline	7.43	1.44	15.9	66.2	0.00	4.28	3.88	0.0600	0.0200	0.620	0.210
Feldspar	6.77	0.160	13.8	70.4	0.0100	7.88	0.330	0.0600	0.0200	0.260	0.350
Fluorspar tailings	0.160	0.310	3.63	91.8	0.0400	1.10	1.01	0.00	0.0200	0.860	1.10

## Data Availability

The original contributions presented in this study are included in the article. Further inquiries can be directed to the corresponding author.
